# HAX1 regulates E3 ubiquitin ligase activity of cIAPs by promoting their dimerization

**DOI:** 10.18632/oncotarget.2459

**Published:** 2014-09-29

**Authors:** Jin Sun Choi, Byoung Chul Park, Seung Wook Chi, Kwang-Hee Bae, Sunhong Kim, Sayeon Cho, Woo-Chan Son, Pyung Keun Myung, Jeong-Hoon Kim, Sung Goo Park

**Affiliations:** ^1^ Medical Proteomics Research Center, Korea Research Institute of Bioscience & Biotechnology (KRIBB), Daejeon, Republic of Korea; ^2^ Cell Function Regulation Research Center, Korea Research Institute of Bioscience & Biotechnology (KRIBB), Daejeon, Republic of Korea; ^3^ Targeted Medicine Research Center, Korea Research Institute of Bioscience & Biotechnology (KRIBB), Daejeon, Republic of Korea; ^4^ Targeted Gene Regulation Research Center, Korea Research Institute of Bioscience & Biotechnology (KRIBB), Daejeon, Republic of Korea; ^5^ College of Pharmacy, Chung-Ang University, Seoul, Republic of Korea; ^6^ Asan Institute for Life Sciences and Asan Medical Center, Seoul, Republic of Korea; ^7^ Department of Pathology, University of Ulsan College of Medicine, Asan Medical Center, Seoul, Republic of Korea; ^8^ College of Pharmacy, Chungnam National University, Daejeon, Republic of Korea; ^9^ Department of Functional Genomics, Daejeon, Republic of Korea; ^10^ Department of Biomolecular Science, University of Science and Technology (UST), Daejeon, Republic of Korea

## Abstract

HS-1-associated protein X-1 (HAX1) is a multi-functional protein which was first identified as a Hematopoietic cell specific Lyn Substrate 1 (HS1)-binding protein. Although the roles of HAX1 in apoptosis have been unraveled and HAX1 has been proposed to be involved in several diseases, additional roles of HAX1 are still being identified. Here, we demonstrated that HAX1 directly interacted with cellular Inhibitor of Apoptosis Proteins (cIAPs), ubiquitin E3 ligases which regulate the abundance of cellular proteins, via ubiquitin-dependent proteasomal degradation. We showed that HAX1 promotes auto-ubiquitination and degradation of cIAPs by facilitating the intermolecular homodimerization of RING finger domain. Moreover, HAX1 regulates the non-canonical Nuclear Factor-κB (NF-κB) signaling pathway by modulating the stability of NF-κB-Inducing Kinase (NIK), which is one of the substrates of cIAPs. Taken together, these results unveil a novel role of HAX1 in the non-canonical NF-κB pathway, and provide an important clue that HAX1 is a potential therapeutic target for the treatment of cancer.

## INTRODUCTION

HS-1-associated protein X-1(HAX1) is a ubiquitously expressed, multifaceted protein with multiple protein-protein interaction domains [1]. It was initially observed in mitochondria, while later it was also found to be localized in other cellular compartments [1, 2]. HAX1 exhibits weak sequence homology with the anti-apoptotic protein, B cell/lymphoma/leukemia-2 (Bcl-2), containing an N-terminal acidic domain, putative Bcl-2 homology domains (BH1 and BH2), a PEST motif, and a predicted C-terminal transmembrane domain [1]. Reminiscent to the roles of members of the Bcl-2 family, HAX1 prevents apoptosis by directly inhibiting the processing of caspase 9 in cardiac myocytes [3] or by modulating Ca^2+^ homeostasis via interacting with phospholamban (PLN) and sarcoplasmic reticulum Ca^2+^ ATPase (SERCA2) [4, 5]. Another mechanism by which HAX1 prevents apoptosis is that HAX1 contributes to presenilin-associated, rhomboid-like-mediated processing and activation of the serine protease HtrA2, thereby preventing accumulation of pro-apoptotic Bax in the outer mitochondrial membrane [6]. Consistent with this anti-apoptotic role, the level of HAX1 is elevated in various metastatic cell lines, including leukemia, melanoma, breast, lung and lymphoma [7]. Moreover, it has been known that homozygous mutations in HAX1 gene cause neutrophil apoptosis, resulting in autosomal recessive severe neutropenia in human [8, 9]. Homozygous null mice for HAX1 recapitulate the phenotype of human neutropenia, leading to postnatal lethality [6].

Inhibitor of apoptosis (IAP) protein family is an endogenous and negative regulator of apoptosis through the direct inhibition of caspases and/or the suppression of apoptotic signaling pathways [10–12]. This family includes X-linked inhibitor of apoptosis protein (XIAP), cellular inhibitor of apoptosis 1 and 2 (cIAP1, cIAP2), melanoma inhibitor of apoptosis (ML-IAP) and survivin [10]. Numerous studies have shown that IAPs are overexpressed in several types of cancer cells [13–15] and that elevated IAP levels contribute to the resistance to cytotoxic therapies, indicating that IAPs are important therapeutic targets for cancer treatment [16, 17]. Among the IAP family members, cIAP1 and cIAP2 inhibit apoptosis by suppressing the apoptotic signaling pathway rather than by directly inhibiting caspase activity [18]. One of the interesting features of cIAP1 and cIAP2 is that they can act as an E3 ubiquitin ligases with their own RING finger domain [19]. Under unstimulated conditions, cIAPs together with tumor necrosis factor (TNF)-associated factors 1 and 2 (TRAF1 and TRAF2), ubiquitinate nuclear factor kappa B (NF-κB)-inducing kinase (NIK), which results in the degradation of NIK through the proteasomal pathway [20, 21]. Once the non-canonical NF-κB signaling pathway is activated by treatment with a set of ligands that belong to the TNFα superfamily, including CD40 ligand (CD40L) [22], B cell activating factor (BAFF) [23], and lymphotoxin-related inducible ligand that competes for glycoprotein D binding to herpesvirus entry mediator on T cells (LIGHT) [24], complexes containing cIAPs are recruited to the receptors, resulting in the stabilization of NIK, which subsequently phosphorylates and activates IκB Kinase α (IKKα) [25]. Next, activated IKKα phosphorylates p100/NF-κB2 and induces partial degradation of p100 to p52 [26]. The N-terminal baculovirus IAP repeat (BIR) domains of IAP family members have been demonstrated to bind to Smac/DIABLO (second mitochondrion-derived activator of caspase) protein, which induces rapid proteasomal degradation of cIAPs [27, 28]. In addition, cIAP1/2 also contain an ubiquitin-associated (UBA) domain that binds to polyubiquitin chains [29] and a less well characterized caspase-recruitment domain (CARD), which was found to be an intrinsic inhibitory domain. Recent studies have demonstrated that Smac mimetics (SMs), which are synthetic compounds and resemble the interacting domain of Smac to cIAPs, bind to BIR2 and BIR3 domains of cIAPs [30] and induce auto-ubiquitination followed by the degradation of cIAPs by promoting the dimerization of RING finger domain [31, 32]. Although numerous studies demonstrated that SMs induce degradation of cIAPs by facilitating their RING dimerization, a cellular counterpart of SMs has not been identified yet.

Here, we show that HAX1 binds to cIAPs *in vitro* and *in vivo*, and this interaction promotes auto-ubiquitination of cIAPs by promoting dimerization of cIAPs. Furthermore, we demonstrate that HAX1 regulates the non-canonical NF-κB pathway through stabilization of NIK. These results reveal a novel role of HAX1 in non-canonical NF-κB signaling pathway.

## RESULTS

### HAX1 interacts with cIAPs *in vivo* and *in vitro*

**Figure 1:**
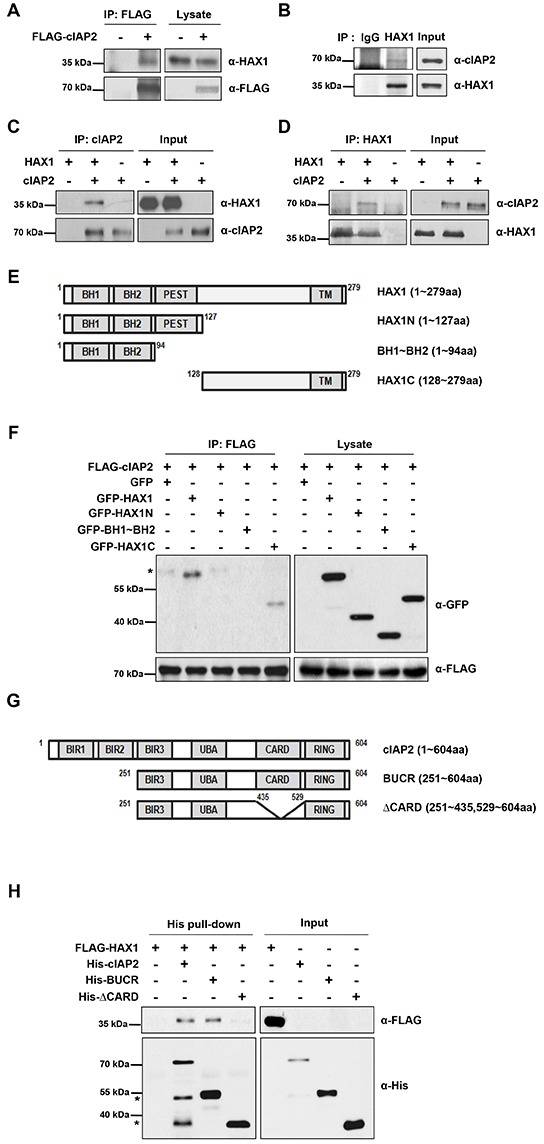
HAX1 interacts with cIAP2 **(A)** FLAG-cIAP2 protein was expressed in HEK293T cells and immunoprecipitated with anti-FLAG M2 antibody-conjugated agarose beads. The immune complexes were analyzed by immunoblotting with anti-HAX1 antibody and anti-FLAG antibody. **(B)** The lysates from MDA-MB-231 cells were incubated with IgG or anti-HAX1 antibody, and the immune complexes were analyzed by immunoblotting with anti-cIAP2 antibody and anti-HAX1 antibody. **(C,D)** Mixtures containing purified HAX1 protein or/and cIAP2 protein were immunoprecipitated with anti-cIAP2 antibody **(C)** or anti-HAX1 antibody **(D)**, and then the immune complexes were analyzed by immunoblotting with anti-cIAP2 antibody and anti-HAX1 antibody. **(E)** Schematic diagram of various HAX1 deletion mutants. BH; Bcl-2 homology domain, PEST; proline (P), glutamic acid (E), serine (S), and threonine (T)-enriched sequence domain, TM; transmembrane domain. **(F)** FLAG-cIAP2 protein was expressed with various GFP-HAX1 deletion mutants in HEK 293T cells. FLAG-cIAP2 was precipitated with anti-FALG M2 antibody-conjugated agarose beads and then anti-GFP antibody and anti-FLAG antibody were used to detect HAX-1 mutants and cIAP2. Asterisk indicates nonspecific bands. **(G)** Schematic diagram of various cIAP2 deletion mutants. **(H)** After purified FLAG-HAX1 protein was incubated with various His-cIAP2 proteins, His-cIAP2 proteins were subjected to Ni-NTA column to pull down the protein complexes. The resulting complexes were analyzed by immunoblotting with anti-His and anti-FLAG antibodies. Asterisks indicate nonspecific bands.

In the previous study, we found that XIAP interacts with HAX1 *in vitro* and *in vivo* [33]. Because XIAP is a member of the IAP family proteins, we hypothesized that cIAP would also interact with the HAX1 protein. To test this hypothesis, we performed co-immunoprecipitation experiments in HEK293T cells. Exogenously expressed FLAG-cIAP2 or HA-cIAP1 was immunoprecipitated and the resulting immune complexes were analyzed by immunoblotting with anti-HAX1 antibody, anti-HA antibody, and anti-FLAG antibody. Interestingly, it was found that FLAG-cIAP2 or HA-cIAP1 interacted with endogenous or exogenous HAX1, respectively (Figure 1A and Supplementary Figure S1A). To corroborate this interaction in physiological condition, we performed co-immunoprecipitation experiment and proximity ligation assay in endogenous level. First, endogenous cIAP2 was co-immunoprecipitated with endogenous HAX1 in MDA-MB-231 cell line (Figure 1B). Furthermore, using proximity ligation assay technique, we showed that endogenous HAX1 is localized in the vicinity of cIAP1 and cIAP2 (Supplementary Figure S2).

In order to determine if cIAP2 directly bind to HAX1 or not, an *in vitro* binding assay was carried out using recombinant cIAP2 and HAX1 proteins. As a result, recombinant HAX1 proteins were co-immunoprecipitated with cIAP2 (Figure 1C) and vice versa (Figure 1D), clearly demonstrating that cIAP2 directly binds to HAX1. To further characterize the region responsible for the interaction, various truncation mutants of HAX1 and cIAP2 were generated (Figure 1E and G). Co-immunoprecipitation experiment in HEK293T cells revealed that C-terminus of HAX1 (amino acid 128–279) binds to cIAP2 (Figure 1F). Meanwhile, the *in vitro* binding assay using truncated cIAP2 and purified HAX1 proteins indicated that the CARD domain of cIAP2 is required for the interaction with HAX1 (Figure 1H). In addition, co-immunoprecipitation experiment in HEK293T cells suggested that the CARD domain is sufficient for this interaction (Supplementary Figure S1B). Taken together, these results showed that the C-terminal region of HAX1 interacts with the CARD domain of cIAP2.

### HAX1 promotes degradation of cIAP2

**Figure 2:**
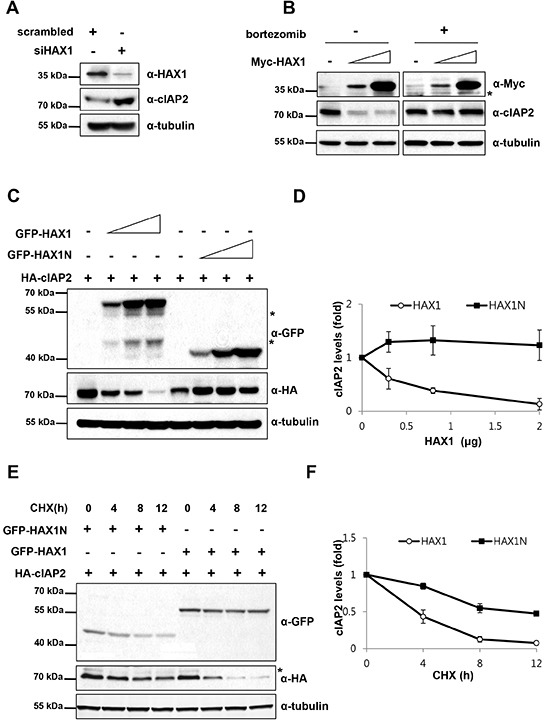
HAX1 promotes degradation of cIAP2 **(A)** MDA-MB-231 cells were transfected with siHAX1 or scrambled control for 24 h and the cell lysates were analyzed by immunoblotting using anti-HAX1, anti-cIAP2, and anti-tubulin antibodies. **(B)** MDA-MB-231 cells were transfected with increasing amounts of Myc-HAX1 (0, 0.8, and 2 μg). After 24 h, the cells were treated with or without a proteasome inhibitor, bortezomib (10 μM), for an additional 7 h. The cell lysates were then examined by immunoblot analysis with anti-FLAG, anti-Myc, and anti-tubulin antibodies. Asterisk indicates nonspecific bands. **(C)** HEK 293T cells were co-transfected with HA-cIAP2 and increasing amounts of GFP-HAX1 or HAX1N. The cell lysates were subjected to immunoblot analysis using appropriate antibodies. Asterisks indicate nonspecific bands. **(D)** Quantitation of the results from (C). Data are presented as mean ± SEM (error bars) of three independent experiments. **(E)** HEK 293T cells were co-transfected with HA-cIAP2 and increasing amounts of GFP-HAX1 or GFP-HAX1N. 24 h after transfection, the cells were treated with a protein synthesis inhibitor, cycloheximide (20 μg/mL), for the indicated time. Then, the prepared cell lysates were subjected to immunoblot analysis using appropriate antibodies. Asterisks indicate nonspecific bands. **(F)** Quantitation of the results from (E). Data are presented as mean ± SEM (error bars) of three independent experiments.

It has been shown that cIAPs regulate the stability of various cellular proteins as well as themselves through their E3 ubiquitin ligase activity [19, 34–36]. Thus, we wondered whether HAX1 regulates stability of cIAP2. To this end, we examined the protein level of cIAP2 when the level of HAX1 protein was altered. Compared with the scrambled control, the reduced expression of HAX1 by siRNA increased the level of endogenous and transfected cIAPs (Figure 2A and Supplementary Figure 3A for endogenous cIAPs, Supplementary Figure S3B and S4A for overexpressed cIAPs). On the other hand, overexpression of HAX1 reduced the level of endogenous and exogenous cIAPs in a dose-dependent manner (Figure 2B; left panels, Supplementary Figure S3C and S4B). However, the proteasome inhibitor, bortezomib, completely blocked the effect of HAX1 on cIAP2 (Figure 2B; right panels), implying that HAX1 promotes the proteasomal degradation of cIAP2. In addition, overexpression of HAX1N, which does not bind to cIAP2 (Figure 1F), has no effect on the stability of cIAP2, indicating that the degradation of cIAP2 is mediated by the physical interaction between cIAP2 and HAX1 (Figure 2C and D, Supplementary Figure S4C). As HAX1 has been demonstrated to bind to some mRNA and attenuate their processing [37, 38], mRNA levels of cIAP1 and cIAP2 were examined by RT-qPCR to see if HAX1 affects mRNAs of cIAPs. The transfection of siRNA of HAX1 did not change the level of mRNA of cIAPs (Supplementary Figure S4D), indicating that HAX1 regulates cIAPs post transcriptionally. Moreover, we also monitored the half-life of cIAP2 protein in the presence of the protein synthesis inhibitor, cycloheximide (CHX). Overexpression of full-length HAX1 induced more rapid degradation of cIAP2 by two fold than HAX1N did (Figure 2E and F). Collectively, these results demonstrate that HAX1 promotes the degradation of cIAP1 and cIAP2.

### HAX1 augments the E3 ubiquitin ligase activity of cIAP2

**Figure 3:**
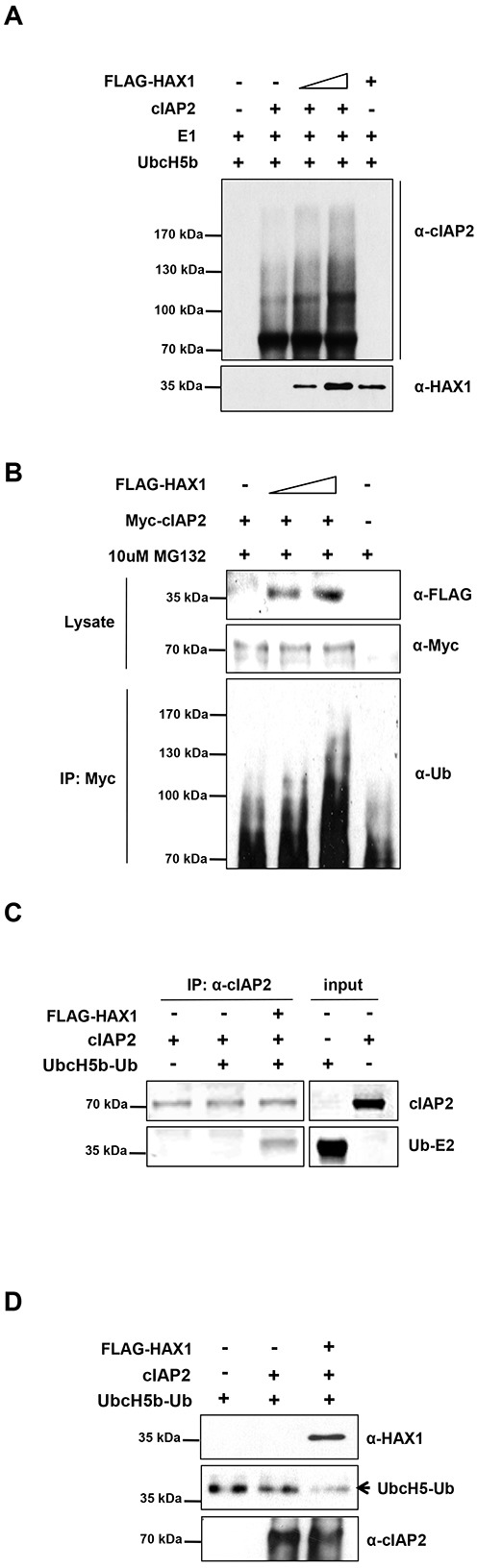
HAX1 augments the E3 ligase activity of cIAP2 **(A)** Purified cIAP2 protein was pre-incubated with increasing amounts of HAX1 proteins and the protein mixture were then incubated with E1, UbcH5b, ubiquitin, and ATP at 37°C for 1 h. The reaction mixtures were analyzed by immunoblotting using an anti-cIAP2 antibody and anti-HAX1 antibody. **(B)** HEK 293T cells were co-transfected with Myc-cIAP2 and increasing amounts of FLAG-HAX1. After 24 h, the cells were treated with a proteasome inhibitor, MG132 (10 μM), for an additional 7 h. Myc-cIAP2 was precipitated with anti-Myc antibody-conjugated agarose beads and the resulting complexes were analyzed by immunoblotting using an anti-ubiquitin antibody (bottom panel). The expression level of each plasmid was determined using anti-FLAG and anti-Myc antibodies (top two panels). **(C)** Purified cIAP2 protein was pre-incubated with or without HAX1 protein and the protein mixtures were then incubated with UbcH5b-Ub at 4°C for 1 h. The reaction mixtures were immunoprecipitated with an anti-cIAP2 antibody and immune complexes were then separated by SDS-PAGE. **(D)** Purified cIAP2 was pre-incubated with or without HAX1 protein and the protein mixtures were then incubated with UbcH5b-Ub at 37°C for the 6 h. The resulting reaction mixtures were subjected to immunoblot using the indicated antibodies to analyze the conversion of the UbcH5b-Ub to free UbcH5b.

Recent reports have shown that the abundance of cIAPs is governed by their auto-ubiquitination, which could serve as an indirect measure of E3 activity [19, 35, 39]. Therefore, we decided to investigate whether HAX1 could affect auto-ubiquitination of cIAP2 by *in vitro* auto-ubiquitination assay. As previously reported, recombinant cIAP2 proteins readily produced poly-ubiquitin chains in the presence of E1, UbcH5b, ubiquitin, and ATP (Figure 3A). When the increasing amount of HAX1 proteins was added, the auto-ubiquitination of cIAP2 increased in proportion to amounts of added HAX1 proteins (Figure 3A). However, HAX1N failed to promote the formation of poly-ubiquitin chains, which suggested that the physical interaction between cIAP2 and HAX1 is required (Supplementary Figure S5A). It is of note that the reaction without cIAP2 in the presence of HAX1 failed to produce poly-ubiquitin chains, which excluded the possibility that HAX1 could act as an E3 ligase. To further confirm this in cellular environment, exogenously expressed Myc-cIAP2 was immunoprecipitated and the resulting precipitates were analyzed by immunoblotting using anti-ubiquitin antibody. Ubiquitination of cIAP2 was increased in the presence of HAX1, suggesting that HAX1 promotes auto-ubiquitination of cIAP2 *in vivo* (Figure 3B).

The activity of E3 ubiquitin ligase can be determined biochemically by two methods; one is to measure the interaction between RING finger domains of E3 ligases and ubiquitin conjugated E2 [40, 41] and the other is to measure the ability of E3 ligases to liberate ubiquitin from ubiquitin conjugated E2 [31, 42]. Therefore, we examined whether HAX1 could influence the ability of cIAP2 to interact with ubiquitin conjugated UbcH5b (UbcH5b-Ub). To this end, UbcH5b-Ub was prepared using E1, UbcH5b, ubiquitin, and ATP (Supplementary Figure S5B and S5C). Next, prepared UbcH5b-Ub and purified cIAP2 were used for the binding assay in the absence or presence of HAX1. The binding affinity of cIAP2 to UbcH5b-Ub strongly increased in the presence of HAX1 (Figure 3C). Furthermore, we monitored the ability of cIAP2 to discharge ubiquitin. The mixtures containing cIAP2 and UbcH5-Ub were incubated with HAX1 or without HAX1 and the resulting mixtures were subjected to immunoblot analyses. Consistent with the result shown in Figure 3C, HAX1 facilitated discharge of ubiquitin from UbcH5b-Ub (Figure 3D). Taken together, these results suggest that HAX1 augments the E3 ligase activity of cIAP2 through physical interaction.

### HAX1 facilitates the dimerization of the cIAP2 RING domain

**Figure 4:**
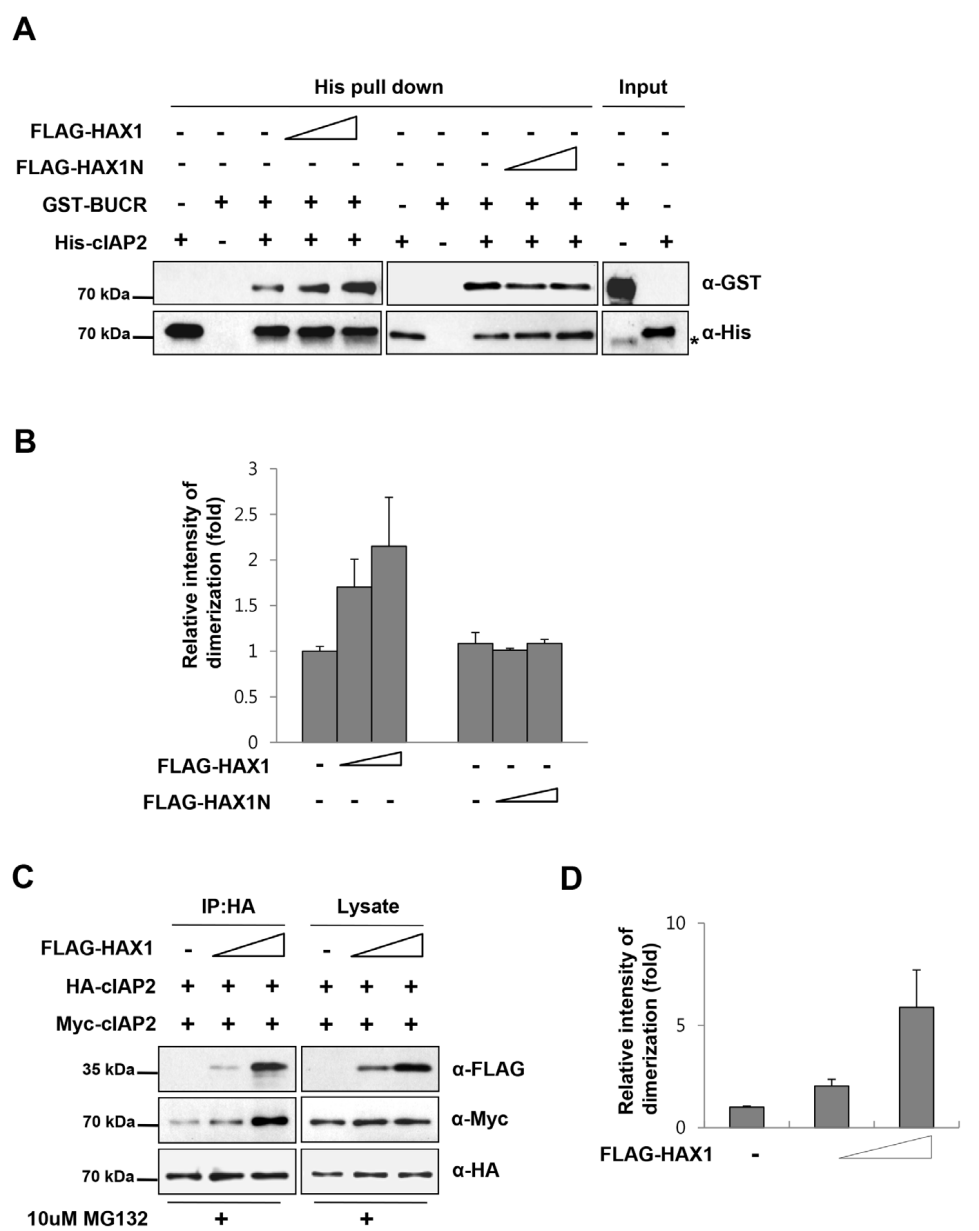
HAX1 facilitates the dimerization of the cIAP2 RING domain **(A)** Mixtures containing purified His-cIAP2 and GST-BUCR proteins were incubated with increasing amounts of HAX1 or HAX1N proteins and then examined by the His-pull down assay. Briefly, proteins were immunoprecipitated with Ni-NTA agarose resin and the resulting complexes were analyzed by immunoblotting with anti-His and anti-GST antibodies. **(B)** Quantitation of the results from (A). Data are presented as mean ± SEM (error bars) of three independent experiments. **(C)** HA-cIAP2 and Myc-cIAP2 proteins were expressed with increasing amounts of FLAG-HAX1 in HEK 293T cells. After 24 h, the cells were treated with or without a proteasome inhibitor, MG132 (10 μM), for an additional 7 h and cell lysates were immunoprecipitated with anti-HA agarose. The resulting complexes were analyzed by immunoblotting using appropriate antibodies. **(D)** Quantitation of the results from (C). Data are presented as mean ± SEM (error bars) of three independent experiments.

It has been demonstrated that the stable dimer formation of cIAPs via their RING finger domains is prerequisite for their activation and efficient binding to ubiquitin conjugating enzyme E2 [31, 32]. As HAX1 activates the E3 ligase activity of cIAP2, we hypothesized that HAX1 could affect the dimerization of cIAPs. Thus, to test this hypothesis, we performed an *in vitro* dimerization assay using purified proteins. For this purpose, full-length His-tagged cIAP2 (His-cIAP2) and GST-tagged cIAP2 fragment (GST-BUCR, Figure 1G) were used because the BUCR domain of cIAP was sufficient for the dimerization [31]. When increasing amounts of full length HAX1 were added, the degree of interaction between His-cIAP2 and GST-BUCR proportionally increased; however HAX1N had no effect (Figure 4A and 4B). To further confirm this observation *in vivo*, two differently tagged cIAP2 plasmids (HA-cIAP2 and Myc-cIAP2) were expressed together with or without HAX1 and the degree of dimer formation of these two cIAPs was analyzed by co-immunoprecipitation. In consistent with the *in vitro* results obtained in Figure 4A and 4B, overexpression of HAX1 augmented the dimer formation of cIAP2 (Figure 4C and 4D). Collectively, these data suggests that HAX1 facilitates the dimerization of cIAP2.

### HAX1 regulates the degradation of NIK

**Figure 5:**
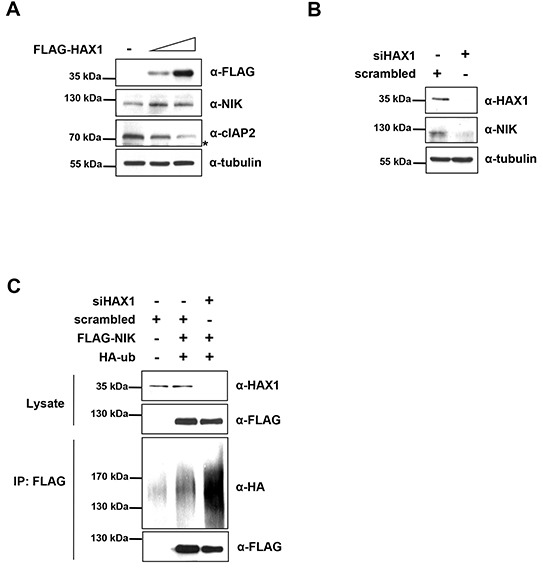
HAX1 regulates the degradation of NIK **(A)** MDA-MB-231 cells were transfected with increasing amounts of FLAG-HAX1 (0, 0.8, and 2 μg) for 24 h, and the cell lysates were then examined by immunoblotting using appropriate antibodies. Asterisk indicates nonspecific bands. **(B)** MDA-MB-231 cells were transfected with siHAX1 or scrambled control for 24 h and the cell lysates were analyzed by immunoblotting using anti-HAX1, anti-NIK, and anti-tubulin antibodies. **(C)** HEK 293T cells were co-transfected with siHAX1 or scrambled control and FLAG-NIK and HA-ubiquitin. After 24 h, the cell lysates were immunoprecipitated with anti-FLAG M2 antibody-conjugated agarose beads and the resulting complexes were analyzed by immunoblotting with anti-HA and anti-FLAG antibodies (bottom two panel). The expression level was determined using anti-HAX1 and anti-FLAG antibodies (top two panels).

Because HAX1 regulates the level of cIAP2, which in turn modulates the abundance of its substrates, we then monitored the level of a well-known substrate of cIAP2. In particular, NF-κB-inducing kinase (NIK), a substrate of cIAPs, has been demonstrated to be a key regulator of the non-canonical NF-κB pathway [20, 21, 43]. Thus we investigated whether HAX1 would regulate the levels of NIK or not. Overexpression of HAX1 increased the level of endogenous NIK, whereas the cIAP2 level decreased in a dose-dependent manner in MDA-MB-231 cells (Figure 5A). Next, we examined the abundance of NIK by utilizing RNA interference to knock down endogenous HAX1. Compared with scrambled control, knocking down HAX1 with siHAX1 reduced the levels of endogenous and exogenous NIK in MDA-MB-231 cells (Figure 5B and Supplementary Figure S6A). Along these lines, we also investigated the degree of poly-ubiquitination of NIK. Compared with the scrambled control, the amount of poly-ubiquitined NIK increased when cells were treated with siHAX1 (Figure 5C), implying that HAX1 suppresses the ubiquitination of NIK. Our results strongly suggest that HAX1 induces the stabilization of NIK through the degradation of cIAP2.

### HAX1 modulates the non-canonical NF-κB signaling pathway by stabilization of NIK

**Figure 6:**
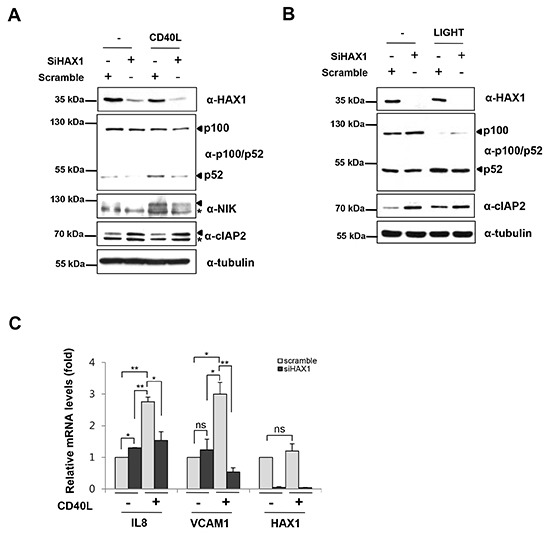
HAX1 modulates the non-canonical NF-κB signaling pathway by stabilization of NIK **(A, B)** MDA-MB-231 cells were transfected with siHAX1 or scrambled control. After 24 h, the cells were stimulated with or without CD40L (1 μg/ml) **(A)** or LIGHT (1 μg/ml) **(B)** for an additional 4 h, and the cell lysates were then examined by immunoblotting using appropriate antibodies. Arrows indicate p100, p52, NIK, and cIAP2 and asterisks indicate nonspecific bands. **(C)** MDA-MB-231 cells were transfected with siHAX1 or scrambled control. After 24 h, the cells were stimulated with or without CD40L (1 μg/ml) (A) for an additional 4 h. Total RNA was isolated from cells, reverse-transcribed, and analyzed by real-time qPCR to determine the mRNA levels of IL-8, VCAM1, and HAX1. Data are presented as mean ± SEM (error bars) of three independent experiments.

The non-canonical NF-κB pathway requires NIK, which phosphorylates and activates IKKα to induce p100 processing to p52 [25, 26]. This, in turn, results in translocation of the p52-RelB complex to the nucleus [44]. Our results consistently demonstrated that HAX1 promotes the degradation of cIAP2 by facilitating its dimerization, which stabilizes NIK. If this is true, it is likely that HAX1 enhances the non-canonical NF-κB signaling pathway, which can be activated by TNFα superfamily ligands, such as CD40L, BAFF, and LIGHT. Thus, we next examined whether siHAX1 affects the non-canonical NF-κB pathway in MDA-MB-231 cells known to be responsive to CD40L, BAFF or LIGHT (Supplementary Figure S7A). Compared with the scrambled control, the reduction of HAX1 expression by siHAX1 led to the stabilization of cIAP2, degradation of NIK, and subsequent inhibition of p100 processing to p52 (Figure 6A and 6B). When the cells were treated with CD40L or LIGHT, the increased p100 processing was hampered by the knockdown of HAX1 (Figure 6A and 6B). We then examined whether HAX1 regulates the transcription of NF-κB target genes upon CD40L or LIGHT stimulation in MDA-MB-231 cells using RT-qPCR. Consistent with the above results, the augmented expression of non-canonical NF-κB target genes, such as interleukin 8 (IL8) and vascular cell adhesion molecule 1 (VCAM1) following stimulation with CD40L or LIGHT, was almost abrogated by HAX1 knockdown (Figure 6C and Supplementary Figure S7B). Collectively, these findings indicate that HAX1 modulates the non-canonical NF-κB signaling pathway by regulating the degradation of NIK and cIAP2.

## DISCUSSION

We have previously demonstrated that HAX1 interacts with BIR2/3 domains of XIAP and suppresses its poly-ubiqutination. However in the present study, we show that HAX1 directly binds to CARD domain of cIAPs and this interaction leads to cIAPs auto-ubiquitination by facilitating their dimerization. This may come from the structural differences between XIAP and cIAPs. XIAP does not possess CARD domain [10, 45] and forms a stable dimer with its RING domain [46]. However cIAPs require induced RING dimerization for its activity because the RING domain of cIAPs is sequestered by BIR and CARD domains [47]. Intriguingly, HAX1 can also form a dimer through its C-terminus region that interacts with both IAP proteins [48]. Based on these data, it may be a plausible model that dimerization of HAX1 induces the dimerization of RING domain of cIAPs by interacting with their CARD domain. The physical interaction of HAX1 with the CARD domain seems to induce a conformational change which renders cIAPs expose their RING domain and thereby facilitates the dimerization of cIAPs. This model is supported by the previous study that the cIAP1^ΔCARD^ mutant readily forms dimers and shows enhanced E3 ligase activity [47]. In case of XIAP, the dimerization of HAX1 may not influence the degree of XIAP RING dimerization since XIAP already exists as a dimer.

Similar to the action of HAX1, the Smac/DIABLO proteins and Smac mimetic compounds (SM) have been shown to induce the dimer formation of RING finger domain of cIAPs, promote auto-ubiquitination/degradation, and subsequently induce caspase-dependent apoptosis in some cancers [31, 32]. However, HAX1 has been known as an anti-apoptotic factor, preventing cell death by various mechanisms [3, 4, 6]. In other words, both SM and HAX1 induce the dimerization and auto-ubiquitination of cIAPs, but produce the contradictory physiological effects. This discrepancy might be explained by our observation that HAX1 suppresses the canonical NF-κB signaling pathway through unidentified mechanisms (unpublished data). Fulda and his colleagues reported that death receptor 5 (DR5), the canonical NF-κB target gene is critical in SM-induced apoptosis [49]. However, we observed that overexpression of HAX1 led to a decreased level of phosphorylated IKKα/β and an increase in the level of IκBα. Furthermore, the transcriptional activity of the canonical NF-κB was inhibited by HAX1 expression (unpublished data). In addition, it has been reported that prolonged SM treatment induces stabilization of NIK, resulting in activation of the non-canonical NF-κB signaling pathway, thereby inducing the expression of the non-canonical NF-κB target gene cIAP2. Because cIAP1 is required for the degradation of cIAP2 and cIAP1 has been already degraded by treatment with SM, prolonged SM treatment leads to increased cIAP2 level in some cancer cells [50, 51]. However, we consistently observed that overexpression of HAX1 induces the reduction of cIAP1/2 levels and the downeregulation of HAX1 by siRNA results in increased levels of cIAP1/2, suggesting that cIAP1 is not required for HAX1-mediated cIAP2 degradation. It is of note that Jitkaew et al. suggested that HAX1 is induced by activation of NF-κB and HAX1 *per se* is an NF-κB target gene [52]. We did not, however, observe any significant changes in both mRNA and protein levels of HAX1 upon the treatment with TNFα, CD40L or LIGHT (Supplementary Figure S8), implicating that HAX1 may not be a direct target of NF-κB in our experimental system.

In the physiological point of view, the activation of the non-canonical NF-κB signaling pathway by NIK is essential for the organization of lymphoid tissue; therefore, deregulation of this pathway leads to several human diseases [21]. Interestingly, some studies showed that HAX1 is required to suppress apoptosis in lymphocyte [6, 53] and homozygotic mutations of HAX1 in human caused increased neutrophil apoptosis, resulting in autosomal recessive severe neutropenia [9]. In this respect, our data suggest that HAX1 may modulate the survival of the lymphocytes by regulating the non-canonical NF-κB signaling pathway.

**Figure 7:**
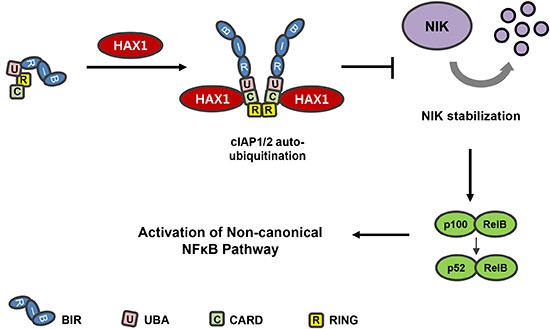
Model depicting the role of HAX1 in the non-canonical NF-κB signaling pathway In the steady state, cIAPs lead to poly-ubiquitination and proteasomal degradation of NIK via their RING domain together with TRAF2 and TRAF3. The degradation of NIK results in the suppression of the non-canonical NF-κB signaling pathway. Binding of HAX1 to the cIAPs results in the auto-ubiquitination and proteasomal degradation of cIAPs. Consequently, reduced cIAP levels leads to the stabilization of NIK, which induces activation of the non-canonical NF-κB signaling pathway.

Collectively, our results demonstrate that interaction between HAX1 and cIAPs promotes auto-ubiquitination and degradation of cIAPs by facilitating their dimerization. Reduced cIAPs levels in turn lead to stabilization of NIK and subsequently activation of the non-canonical NF-κB signaling pathway. The present study provides a mechanism of which HAX1 regulates the non-canonical NF-κB signaling pathway and contributes to the understandings of cIAPs regulation as it closely relates to cancer treatment. The schematic model depicting our results is summarized in Figure 7.

## MATERIALS AND METHODS

### Cell culture and transfections

HEK 293T cells (#CRL-11268) and MDA-MB-231 cells (#HTB-26, ATCC) were cultured in DMEM (GIBCO) supplemented with 10% fetal bovine serum, pen/strep (100 U/mL), and streptomycin (100 U/mL) in humidified incubators at 37°C with 5% CO_2_. Transfection of HEK 293T cells and MDA-MB-231 cells with various expression vectors was carried out using Lipofectamine (Invitrogen) and X-tremeGENE (Roche).

### Expression plasmids

Mammalian expression constructs were obtained as follows. HAX1, cIAP1, cIAP2, and NIK were cloned into the FLAG-tagged vector pFLAG-CMV-2 (Sigma Co., USA) and the Myc-tagged vector pCMV-Myc (Clontech). HAX1, the N-terminal domain (a.a 1–127), the BH1-BH2 domain (a.a 1–94), and the C-terminal domain (a.a 128–279) of HAX1 were cloned into the green fluorescent protein (GFP)-tagged vector pEGFP-C2 (Clontech). Bacterial expression constructs were obtained as follows. cIAP2, BUCT (a.a 251–604), and ΔCARD (a.a 251–435, a.a 529–604) of cIAP2 were cloned into the His-tagged vector pET21a (Merck Millipore) and the GST-tagged vector pGEX-6p-1 (GE Healthcare Life Sciences). The FLAG-N-terminal domain (a.a 1–127) and FLAG-C-terminal domain (a.a 128–279) of HAX1 were cloned into the pET21a vector (Merck Millipore). Baculovirus expression constructs were obtained by inserting FLAG-HAX1 into the pFastBacT^™^ expression vector (Invitrogen).

### Antibodies and reagents

The following antibodies were used for western blot: HAX1 (BD Transduction Laboratories: 610825, 1:500), cIAP2 (ABCAM: ab32059, 1:1000), cIAP1 (R&D Systems: AF8171, 1:500), FLAG (F1804, 1:5000), His (H1029, 1:3000), and α-tubulin (T6074, 1:5000) (Sigma-Aldrich Co. LLC.), p100/p52 (#3017, 1:1000), NIK (#4994, 1:1000), and cIAP2 (#3130, 1:1000) (Cells Signaling Technology, Inc.), GFP (sc-9996, 1:1000) and HA (sc-805, 1:1000) (Santa Cruz Biotechnology, Inc.), and ubiquitin (BML-PW8810, 1:100, Enzo Life Science). Recombinant human CD40 ligand/TNFSF5 (617-CL) and recombinant human LIGHT/TNFSF14 (664-LI) were obtained from R&D Systems. Pierce anti-HA agarose (26181) was obtained from Thermo Scientific. Anti-FLAG M2 affinity gels (A2220), 3× FLAG peptide (F4799), anti-c-Myc agarose affinity gels (A7470), cycloheximide (C4859), and MG132 (C2211) were purchased from Sigma-Aldrich Co. LLC. Bortezomib (velcade) (MG-341) and protein G plus/protein A agarose suspension (IP05) were purchased from Merck Millipore. Complete protease inhibitor cocktail (13760700) was purchased from Roche.

### Immunoprecipitation and western blot analysis

In general, cells were grown in 60-mm plates and then transfected with the designated expression constructs. Subsequently, cells were harvested in 1000 μL lysis buffer (20 mM Tris–HCl [pH 7.5] containing 150 mM NaCl, 1% (v/v) igepal, 10% (w/v) glycerol, and 1 mM complete protease inhibitor cocktail) and incubated at 4°C for 30 min with gentle rocking. Cell lysates were centrifuged at 15,000 × *g* for 15 min to remove cell debris and nuclei. An aliquot (100 μL) of the supernatant was retained for western blot analysis, and the remaining supernatant was subjected to immunoprecipitation. Cell lysates were either mixed with 20 μL of anti-FLAG M2 affinity gel or anti-HA-agarose at 4°C for 4 h with gentle agitation or the lysates were incubated for 2 h at 4°C with appropriate antibodies (2 μg) with gentle rotation. Then, 30 μL of protein G plus/protein A agarose was added to each sample and incubated at 4°C for 2 h with rocking. The antigen–antibody complexes were collected by centrifugation, washed three times with lysis buffer, and then boiled in sodium dodecyl sulfate-polyacrylamide gel electrophoresis (SDS-PAGE) sample buffer (50 μL) for 5 min to elute the antigen complex from the affinity gel. Samples were resolved by SDS-PAGE, transferred to nitrocellulose membranes (10401116, GE Healthcare Life Sciences), and analyzed by immunoblotting using appropriate antibodies. Immunoreactivity was visualized by electrochemiluminescence.

### Bacterial expression and purification of proteins

*Escherichia coli* BL21 (DE3) cells carrying bacterial expression constructs were grown at 37°C and protein expression was induced with 1 mM isopropyl-β-d-thiogalactopyranoside overnight at 18°C. For the purification of His-proteins, cells were washed with cold phosphate-buffered saline (PBS), lysed in lysis buffer (25 mM HEPES [pH 8.0] containing 150 mM NaCl, 0.1% (v/v) igepal, 10% (w/v) glycerol, 5 mM imidazole, 1 mM phenylmethylsulfonyl fluoride, and 1 mM complete protease inhibitor cocktail), sonicated, and spun down. The supernatant was loaded onto an Ni-NTA agarose resin column (70666, Merck Millipore) or pulled down, sequentially washed with 30 bed volumes of buffer (25 mM HEPES [pH 8.0] containing 150 mM NaCl, 0.1% (v/v) igepal, 10% (w/v) glycerol, and 35 mM imidazole), and eluted with 250 mM imidazole, pH 8.0. Similarly, GST-proteins were purified from crude extracts using glutathione-Sepharose 4B (17–0756–01, GE Healthcare Life Sciences) column chromatography. Collected fractions were eluted using the PreScission Protease Cleavage protocol (27–0843–01, GE Healthcare Life Sciences).

### Preparation of recombinant HAX1 in Sf9 cells

To obtain baculovirus-expressed HAX1, recombinant bacmid expressing HAX1 was transfected into Sf9 cells using CellFECTIN^®^ (Invitrogen). The resultant viral pool in the supernatant of the transfected cells was collected 3 days later. Sf9 cells were cultured in Sf-900 II serum-free medium (Invitrogen) at 27°C. To produce recombinant HAX1 protein, Sf9 cells seeded at a density of 1 × 10^6^ cells per mL were infected with recombinant HAX1 virus. The cells were harvested 48 h after infection and cell pellets were resuspended in lysis buffer (20 mM Tris–HCl [pH 7.5] containing 150 mM NaCl, 1% (v/v) igepal, 10% (w/v) glycerol, and 1 mM complete protease inhibitor cocktail). The resuspended cells were lysed using a sonicator. The resulting lysates were centrifuged at 15,000 × *g* for 1 h (twice) and the supernatants were subjected to immunoprecipitation using FLAG M2 affinity gels. Bound proteins were eluted using 3× FLAG peptide.

### siRNA-mediated knockdown

RNA interference was carried out using specific siRNA duplexes to silence HAX1 in HEK 293T or MDA-MB-231 cells. Chemically synthesized siRNA duplexes were purchased from Bioneer Corporation. The following sequences were used for all experiments: 5′-CAGCCCAAAUCCUAUUUCA-3′. Delivery of siHAX1 or scrambled control into the cells was carried out using the Neon^®^ transfection system (Invitrogen) according to the manufacturer’s instructions; cells were then incubated for 24–48 h before analysis.

### *In vitro* binding assay

Purified HAX1 and cIAP2 proteins were preincubated at 4°C for 30 min after which the mixtures were precipitated with protein G plus/protein A agarose and appropriate antibodies in binding buffer (20 mM HEPES containing 100 mM NaCl, 5% [w/v] glycerol, 0.1 mg bovine serum albumin [BSA], and 0.1% Triton X-100) at 4°C for 4 h. The formed immune complexes were then analyzed by immunoblotting using appropriate antibodies.

### *In vitro* auto-ubiquitination assay

The polyubiquitination assay mixture contained 25 mM Tris–HCl, pH 7.4, 5 mM MgCl_2_, 100 mM NaCl, 2 mM ATP, 0.2 mM dithiothreitol (DTT), 20 ng E1, 0.4 μM E2 (UbcH5b, 50 ng), 2 μg ubiquitin,1 μg cIAP2, and HAX1 (0, 0.5, and 5 μg). Reactions were incubated at 37°C for 1 h and the ubiquitinated products were detected by immunoblotting using an anti-cIAP2 antibody.

### *In vitro* binding assays between E2-ubiqutin conjugate and cIAP2 and discharge assay

*In vitro* binding assays between E2-ubiquitin (E2-Ub) conjugate and cIAP2 were performed according to Catherine L. Day’s method [31]. Purified cIAP2 and HAX1 proteins were preincubated at 4°C for 30 min. Then, cIAP2 protein and E2-Ub conjugate were mixed at 4°C for 1 h in PBS buffer containing 0.2% Tween 20 and 2 mM DTT. The reaction mixtures were precipitated with protein G plus/protein A agarose and cIAP2 antibody at 4°C for 4 h and then washed with PBS containing 0.2% Triton X-100 and 2 mM DTT. Bound proteins were eluted with 2× SDS-PAGE sample loading buffer. Samples were resolved by SDS-PAGE, and gels were subsequently stained using the Coomassie Blue staining method. To analyze discharge, purified cIAP2 and E2-Ub conjugate were mixed, HAX1 was added to the mixtures, and the samples were incubated in 50 mM MES, pH 6.5 at 37°C for 6 h. The disappearance of E2-Ub conjugates was analyzed by immunoblotting using an anti-His antibody.

### *In vitro* dimerization assay

Mixtures containing purified His-cIAP2, GST-BUCR proteins, and HAX1 (0, 0.5, and 5 μg) or HAX1N proteins (0, 0.5, and 5 μg) were preincubated at 4°C for 30 min. Next, the reaction mixtures were examined by the His-pull down assay. Briefly, samples were added to Ni-NTA agarose resin in binding buffer (20 mM HEPES containing 100 mM NaCl, 5% [w/v] glycerol, 0.1 mg BSA, and 0.1% Triton X-100), incubated for 4 h at 4°C, and the resulting complexes were analyzed by immunoblotting with anti-His antibody and anti-GST antibody.

### *In vivo* dimerization assay

HEK 293T cells were co-transfected with HA-tagged cIAP2, Myc-tagged cIAP2, and increasing amounts of FLAG-tagged HAX1 (0, 0.8, and 2 μg). After 24 h, the cells were treated with the proteasome inhibitor MG132 (10 μM) for an additional 7 h and immunoprecipitated with anti-HA agarose for 4 h at 4°C. Immune complexes were then analyzed by immunoblotting using appropriate antibodies.

### RNA isolation and RT-qPCR

Total RNA (0.5 μg) isolated using TRIzol (Life Technologies) was reverse-transcribed with SuperScript III (Life Technologies) using random primers. The synthesized complementary DNA was used as template for qPCR using Power SYBR Green Master Mix (Life Technologies) on an Applied Biosystems 7500Fast system (Life Technologies). The primer sequences are presented in Supplementary Table S1. The relative expression level was calculated by the ΔΔCt method using glyceraldehyde-3-phosphate dehydrogenase as internal control. All amplifications were performed in triplicate wells and in more than three independent experiments.

## SUPPLEMENTARY DATA


