# Supplementary material for: HAX1 regulates E3 ubiquitin ligase activity of cIAPs by promoting their dimerization
Source: Oncotarget. 2014 Sep 29;5(20):10084–99. doi: 10.18632/oncotarget.2459 (PMC4259407; doi:10.18632/oncotarget.2459)
Supplement: Supplementary file 1 [file oncotarget-05-10084-s001.pdf]

SUPPLEMENTARY DATA

Proximity ligation assay

Cells were seeded on 35-mm glass-bottom dishes (MatTek Corp., Ashland, MA, USA), and fixed with cold 4% paraformaldehyde for 15 min at room temperature followed by cell-membrane permeabilization with 0.25% Triton-X-100 in PBS for 10 min. The cells were washed three times with PBS, blocked for 1 h at room temperature with 1% BSA in PBS containing and incubated with the indicated antibody pairs in a cell incubator at 37 °C for 2 h. After washing, the slides were incubated with Duolink PLA Rabbit MINUS and PLA Mouse PLUS proximity probes (Olink Bioscience, Uppsala, Sweden) and proximity ligation was performed using the Duolink detection reagent kit (Olink Bioscience) according to the manufacturers protocol. Fluorescence was detected using a Zeiss LSM 510 META confocal microscope (Zeiss, Thornwood, NY, USA). Antibodies used for PLA were: mouse anti-HAX1 (BD Transduction Laboratories: 610825), rabbit anti-cIAP1 (Cell Signaling Technology: #4952), rabbit anti-cIAP2 (ABCAM: ab32059).

E2-ubiquitin conjugate preparation

E2-ubiquitin conjugate preparation was performed according to Catherine L. Day’s method. Purified His-FLAG-E2 (UbcH5b), E1, ubiquitin, and ATP were incubated at 37 °C for 6 h in buffer (25 mM Tris-HCl [pH 7.6] containing 5 mM $MgCl_2$ and 100 mM NaCl).

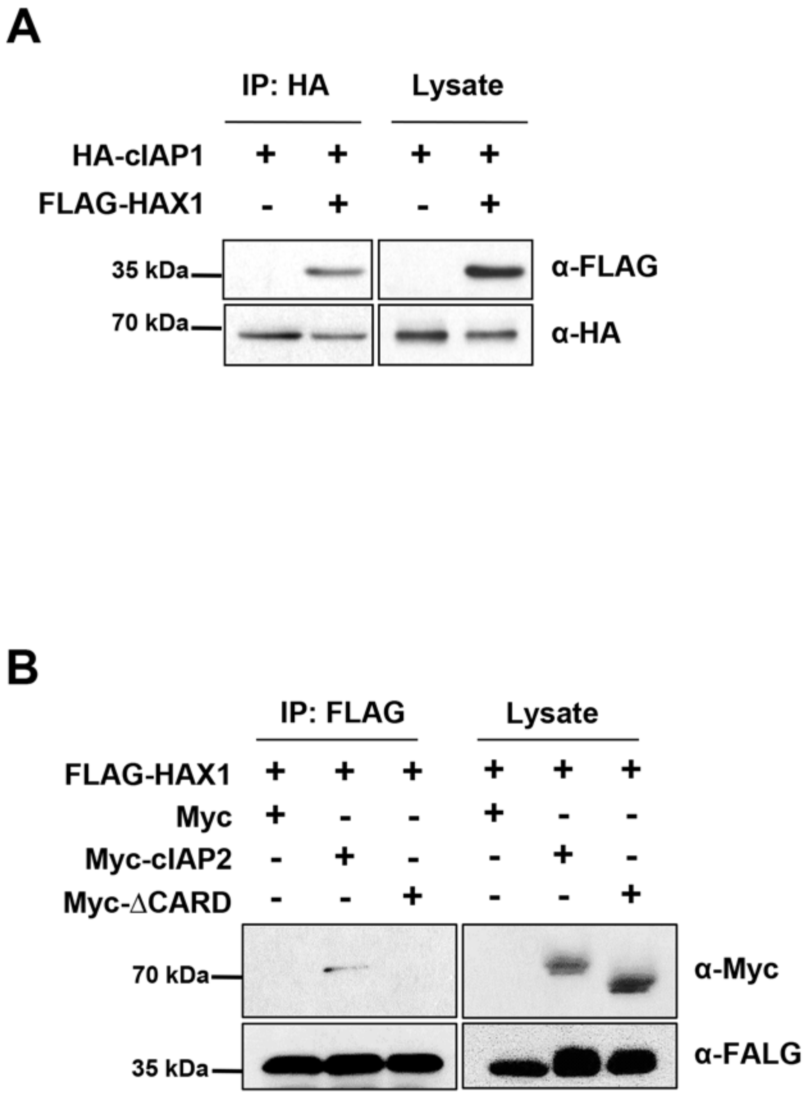

Supplementary Figure S1: HAX1 interacts with cIAPs. (A) HA-cIAP1 and FLAG-HAX1 proteins were expressed in HEK 293T cells and immunoprecipitated with anti-HA agarose. The resulting complexes were analyzed by immunoblotting with anti-FLAG and anti-HA antibodies. **(B)** FLAG-HAX1 protein was expressed with various Myc-cIAP2 deletion mutants in HEK 293T cells. FLAG-HAX1 was precipitated with anti-FALG M2 agarose and then resulting complexes were then analyzed by immunoblotting using anti-Myc antibody and anti-FLAG antibody.

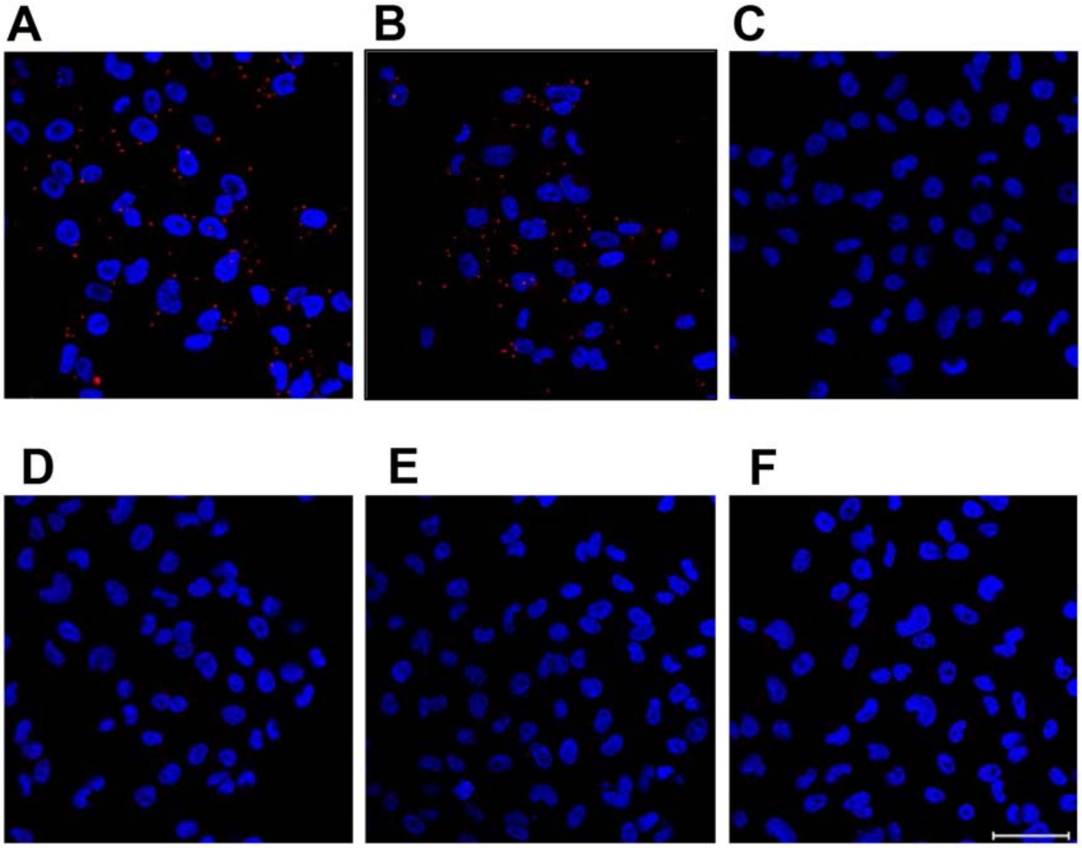

Supplementary Figure S2: Endogenous HAX1 interacts with endogenous cIAPs. Interaction between HAX1 and cIAP2 was examined by the proximity ligation assay. MDA-MB-231 cells were stained with primary antibodies; anti-HAX1 and anti-cIAP1 antibodies **(A)**, anti-HAX1 and anti-cIAP2 antibodies **(B)**, anti-cIAP1 **(C)**, anti-cIAP2 **(D)**, anti-HAX1 **(E)** and without primary antibodies **(F)**, and detected with appropriate combination of PLA probes. PLA signals are shown in red and the nuclei in blue. The scale bar indicates 50 μm.

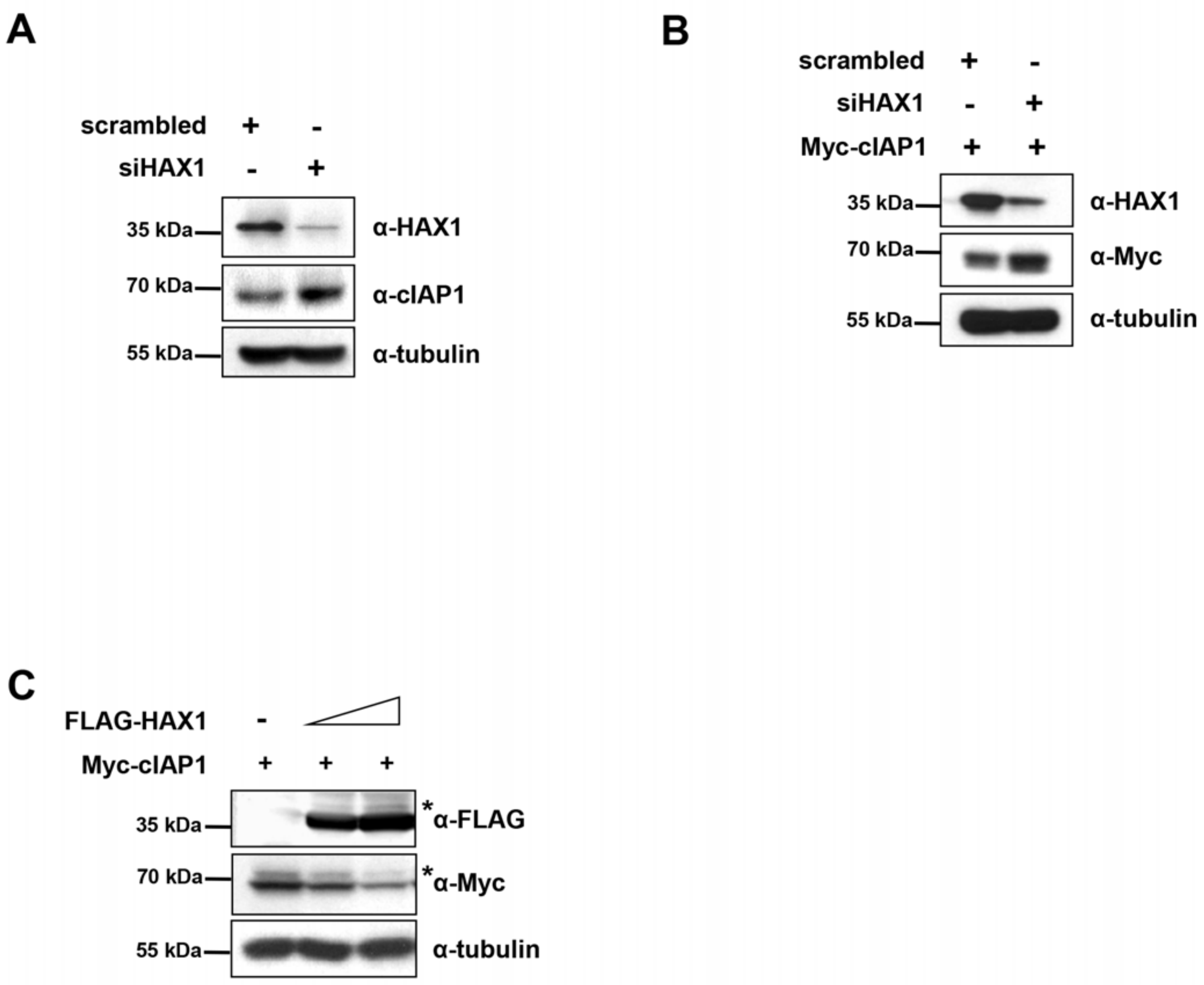

Supplementary Figure S3: HAX1 promotes degradation of cIAP1. **(A)** MDA-MB-231 cells were transfected with siHAX1 or scrambled control for 24 h and cell lysates were analyzed by immunoblotting using anti-HAX1, anti-cIAP1, and anti-tubulin antibodies. **(B)** HEK 293T cells were transfected with siHAX1 or scrambled control and Myc-cIAP1 for 24 h and cell lysates were analyzed by immunoblotting using anti-HAX1, anti-Myc, and anti-tubulin antibodies. **(C)** HEK 293T cells were co-transfected with Myc-cIAP1 and increasing amounts of FLAG-HAX1 (0, 0.8, and 2 μg). After 24 h, the cell lysates were examined by immunoblot analysis with anti-FLAG, anti-Myc, and anti-tubulin antibodies. Asterisks indicate nonspecific bands.

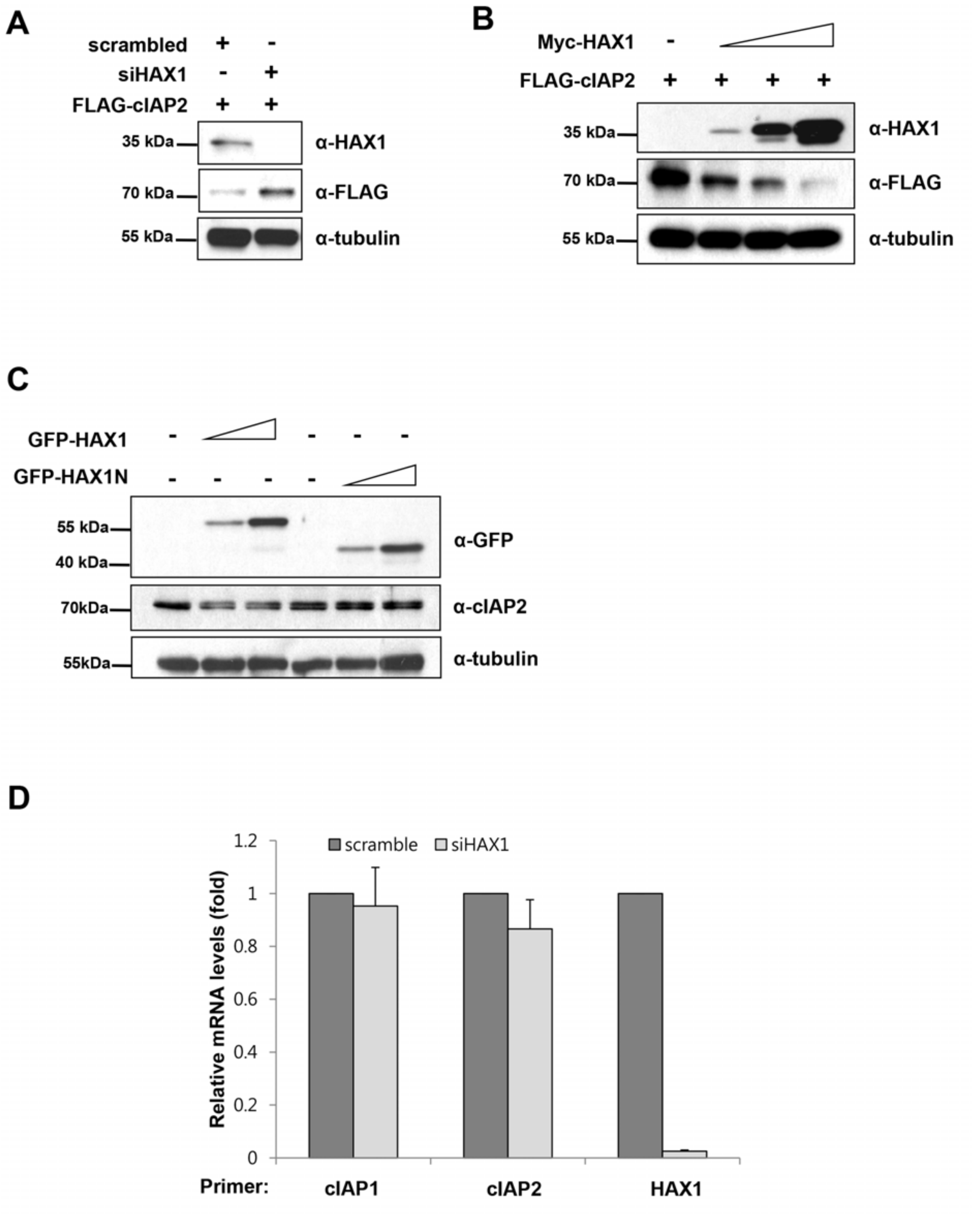

Supplementary Figure S4: HAX1 promotes degradation of exogenous cIAP2. **(A)** HEK 293T cells were transfected with siHAX1 or scrambled control and FLAG-cIAP2 for 24 h and cell lysates were analyzed by immunoblotting using anti-HAX1, anti-FLAG, and anti-tubulin antibodies. **(B)** HEK 293T cells were co-transfected with FLAG-cIAP2 and increasing amounts of Myc-HAX1 (0, 0.8, and 2 μg). After 24 h, the cell lysates were examined by immunoblot analysis with anti-HAX1, anti-FLAG, and anti-tubulin antibodies. **(C)** MDA-MB-231 cells were transfected with increasing amounts of GFP-HAX1 or GFP-HAX1N (0, 0.8, and 2 μg). After 24 h, the cells were examined by immunoblot analysis with anti-GFP, anti-cIAP2, and anti-tubulin antibodies. **(D)** MDA-MB-231 cells were transfected with siHAX1 or scrambled control. After 24 h, RNAs were isolated from cells, reverse-transcribed, and analyzed by real-time qPCR to determine the mRNA levels of cIAP1, cIAP2, and HAX1. Data are presented as mean ± SEM (error bars) of three independent experiments.

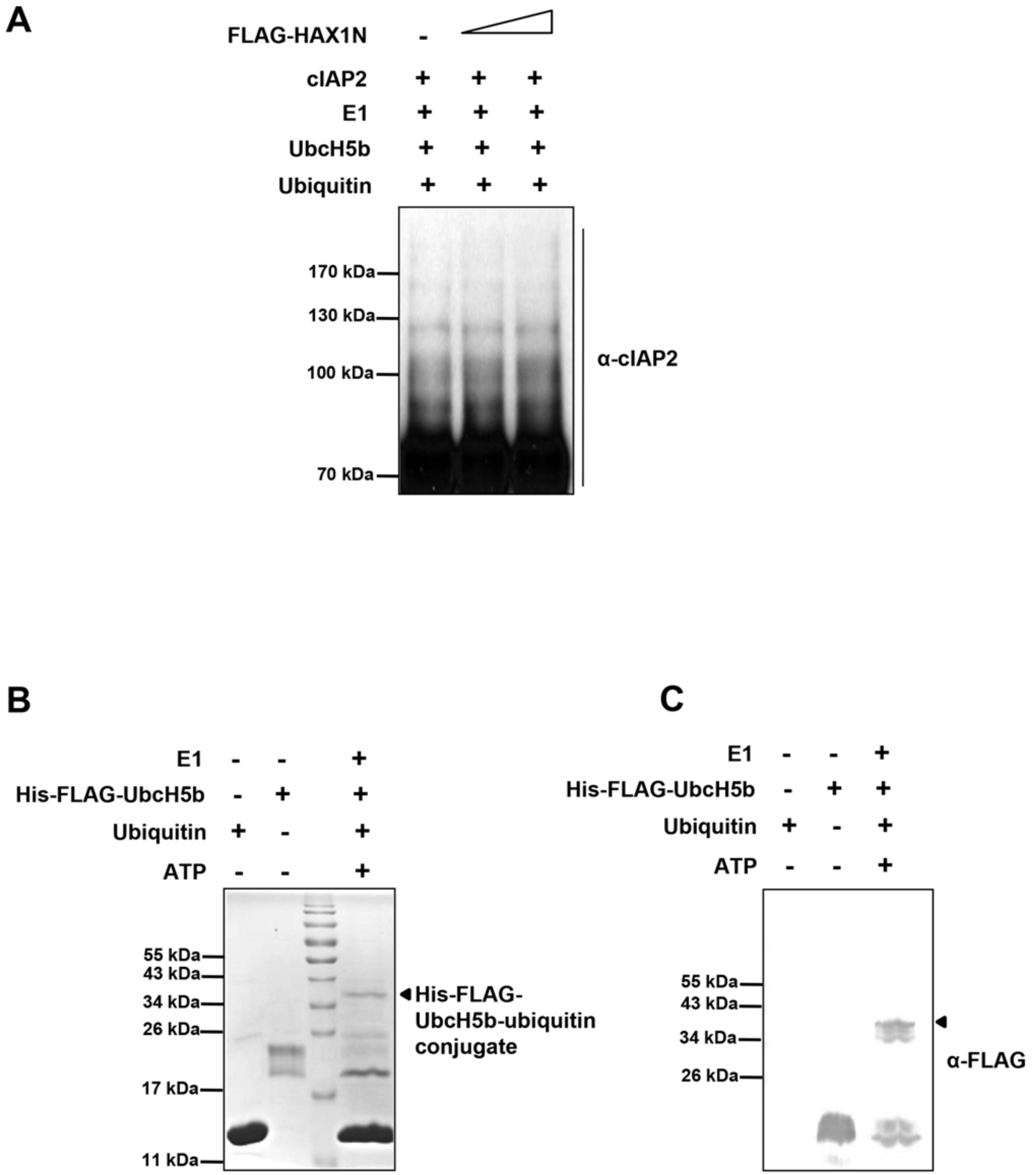

Supplementary Figure S5: HAX1N does not facilitate the auto-ubiqutination of cIAP2. (A) Purified cIAP2 protein was preincubated with increasing amounts of HAX1N proteins and the protein mixture were then incubated with E1, UbcH5b, ubiquitin, and ATP at 37 °C for 1 h. The reaction mixtures were analyzed by immunoblotting using an anti-cIAP2 antibody. **UbcH5-Ub preparation** Purified His-FLAG-UbcH5b, E1, ubiquitin, and ATP were incubated at 37 °C for 6 h until conjugate formation was complete. After incubation, samples were mixed with 2× Tris-glycine gel loading buffer and then separated by SDS-PAGE. UbcH5b-Ub conjugates were visualized by Coomassie Blue staining **(B)** and by immunoblot analysis using an anti-FLAG antibody. Arrows indicate UbcH5b-ubiquitin conjugate **(C)**.

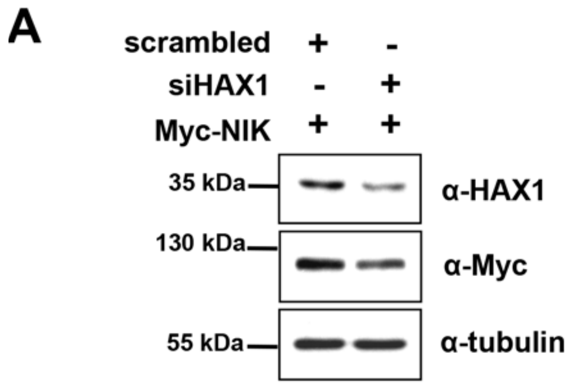

Supplementary Figure S6: siHAX1 promotes the degradation of exogenous NIK. MDA-MB-231 cells were co-transfected with siHAX1 or scrambled control and Myc-NIK for 24 h and the cell lysates were analyzed by immunoblotting using anti-HAX1, anti-Myc, and anti-tubulin antibodies.

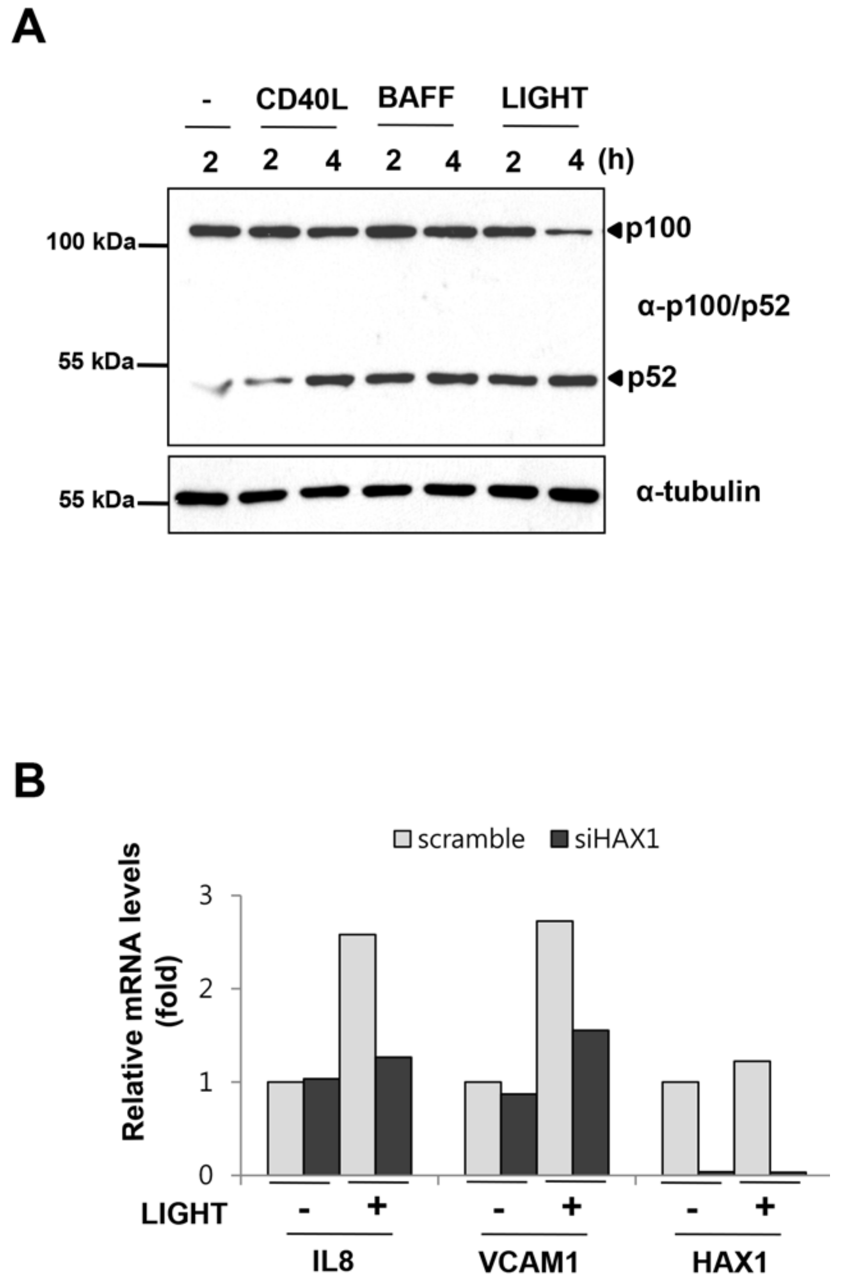

Supplementary Figure S7: siHAX1 decreases mRNA levels of IL-8 and VCAM1 in MDA-MB-231 cells through LIGHT treatment. (A) MDA-MB-231 cells were stimulated with CD40L (1 μg/ml), BAFF (1 μg/ml), and LIGHT (1 μg/ml) for the indicated time and cell lysates were then examined by immunoblotting using anti-p100/p52 and anti-tubulin antibodies. Arrows indicate p100 and p52. **(B)** MDA-MB-231 cells were transfected with siHAX1 or scrambled control. After 24 h, total RNA was isolated from cells, reverse-transcribed, and analyzed by real-time qPCR to determine the mRNA levels of IL-8, VCAM1, and HAX1.

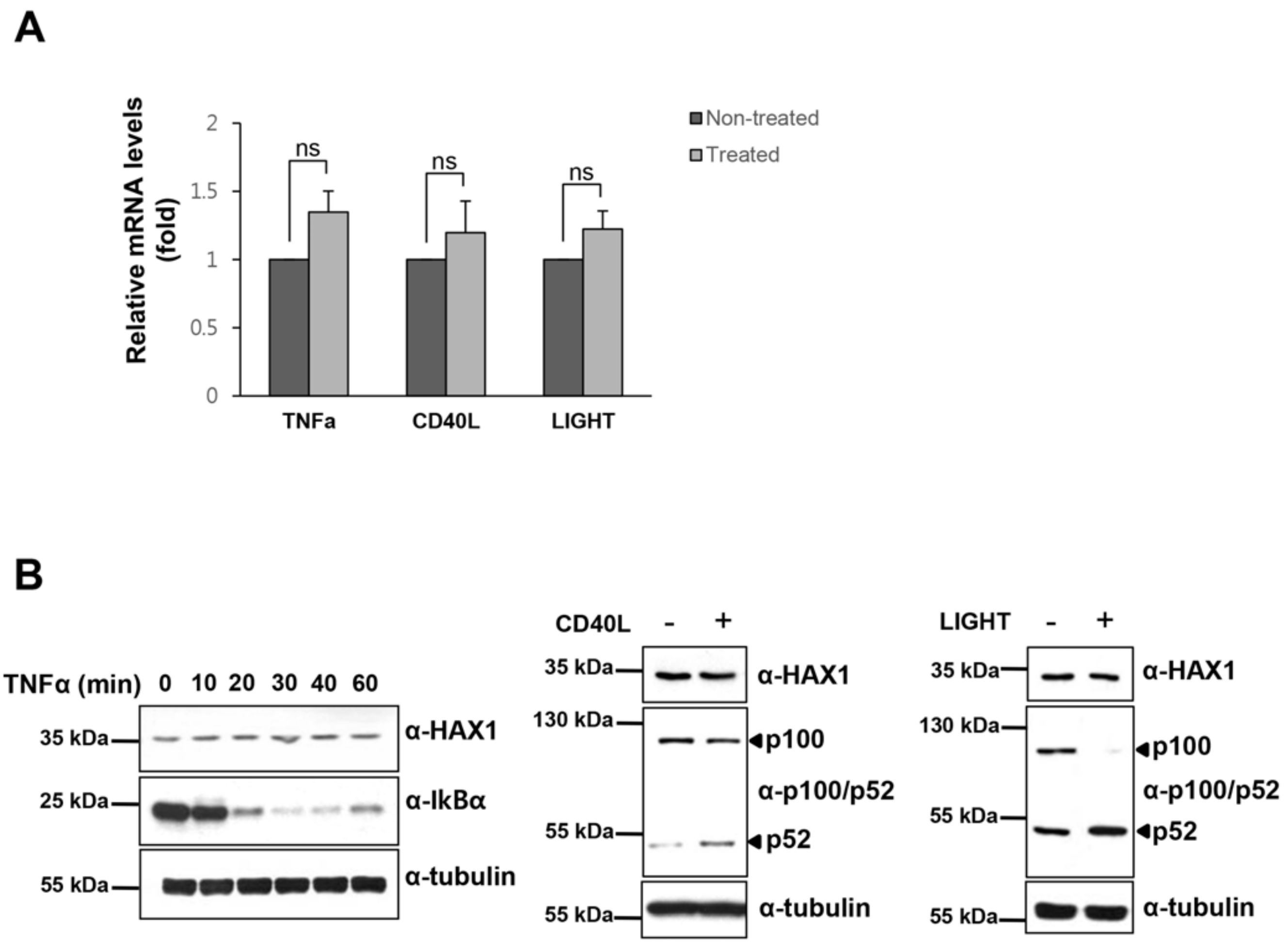

Supplementary Figure S8: HAX1 is not regulated by the canonical/non-canonical NF-κB signaling pathway. (A) HeLa cell was stimulated with TNFα (20 ng/ml) and MDA-MB-231 cells were stimulated with CD40L (1 μg/ml) or LIGHT (1 μg/ml) for 4h and then total RNA was isolated from cells, reverse-transcribed, and analyzed by real-time qPCR to determine the mRNA levels of HAX1. Data are presented as mean ± SEM (error bars) of three independent experiments. $p > 0.5$. **(B)** HeLa cells were treated with TNFα (20 ng/ml) for the indicated times and then analyzed by immunoblotting using anti-HAX1, anti-IκBα, and anti-tubulin antibodies (Left panel). MDA-MB-231 cells were stimulated with or without CD40L (1 μg/ml) (Middle panel) or LIGHT (1 μg/ml) (Right panel) for an additional 4 h, and the cell lysates were then examined by immunoblotting using appropriate antibodies. Arrows indicate p100, p52.

Supplementary Table S1. Primer sequences for quantitative reverse transcription-polymerase chain reaction

For RT-qPCR	Sequence
IL8_q_F	TCTGCAGCTCTGTGTGAAGG
IL8_q_R	ACTTCTCCACAACCCTCTG
VCAM1_q_F	TGTAGTGTCATGGGCTGTGA
VCAM1_q_R	AACTCACAGGGCTCAGGGT
HAX1_q_F	TCCTCCTGAACTTCCAGGTC
HAX1_q_R	GATCCTGGGCTGGTGACTAT
GAPDH_q_F	ACCAGGTGGTCTCCTCTGAC
GAPDH_q_R	TGCTGTAGCCAAATTCGTTG
